# Adolescent health behavior patterns and weight status: a cross-sectional analysis

**DOI:** 10.3389/fpubh.2025.1697321

**Published:** 2025-12-18

**Authors:** Jiayan Gui, Hui Zhang, Jingyou Miao, Xinyao Liu, Lilu Ding, Qiuli Wang

**Affiliations:** 1 Zhejiang Medical & Health Group Quzhou Hospital, Quzhou City, Zhejiang Province, China; 2 Department of Epidemiology and Health Statistics, Hangzhou Medical College, Hangzhou, China

**Keywords:** adolescent health behaviors, latent class analysis (LCA), body weight status, BMI classification, health intervention

## Abstract

**Background:**

Adolescent weight status is shaped by co-occurring behaviors, but variable-centered analyses may obscure heterogeneous patterns. Person-centered approaches can clarify how these patterns relate to BMI.

**Objective:**

To identify adolescent health behavior patterns and assess their associations with BMI categories.

**Methods:**

We conducted a cross-sectional survey of 1,212 students in grades 7–8 from Lin'an District, Hangzhou, China. Six behavior indicators (diet, sugary drinks, outdoor activity, anxiety control, weight-management awareness, and management needs) informed a latent class analysis. Logistic regression, adjusting for demographic and psychosocial factors, estimated associations between class membership and BMI status.

**Results:**

Three health behavior patterns emerged: passive health maintenance (50.9%), self-disciplined health type (32.7%), and high-risk lifestyle (16.5%). Compared to the self-disciplined group, the passive group showed significantly increased risks of overweight (OR = 1.62, 95% CI: 1.02–2.57) and obesity (OR = 1.68, 95% CI: 1.13–2.50), while the high-risk group showed a trend toward increased obesity risk (OR = 1.57, 95% CI: 0.96–2.57, P = 0.072). Female students exhibited lower risks of overweight (OR = 0.56, 95% CI: 0.36–0.87) and obesity (OR = 0.41, 95% CI: 0.28–0.59) compared to males; eighth-grade students had a lower risk of obesity than seventh-grade students (OR = 0.59, 95% CI: 0.40–0.87). Additionally, good sleep quality reduced the likelihood of belonging to the high-risk group (OR = 0.30, 95% CI: 0.17–0.53), and emotional eating increased the risk of being in the passive group (OR = 1.74, 95% CI: 1.31–2.32).

**Conclusions:**

Early adolescents show distinct health behavior patterns with differential weight outcomes. The large passive group, though not overtly high-risk, carries significant overweight risk, highlighting a “moderate-risk blind spot” in weight management. Identifying behavior clusters and tailoring interventions by behavioral profile and sociodemographic context may improve adolescent obesity prevention.

## Introduction

The rising prevalence of overweight and obesity among children and adolescents has become a major public health challenge worldwide. Projections suggest that the number of obese adolescents will increase by 60% over the next decade, reaching 250 million by 2030, thereby placing enormous strain on health systems (1, 2). In China, rapid economic development and lifestyle changes have substantially altered dietary habits and physical activity levels among young people (3, 4). Over the past two decades, the prevalence of overweight and obesity among Chinese adolescents has risen steadily. According to the China National Nutrition and Chronic Disease Report, 19% of children and adolescents aged 7–18 are now classified as overweight or obese, and the growth rate between 2012 and 2020 was more than three times that observed in adults (5).

Adolescent overweight and obesity carry immediate and long-term health consequences. They are associated with poor metabolic profiles, psychosocial difficulties, and increased risks of chronic diseases in adulthood, including cardiovascular disease, a wide range of lifestyle factors contribute to these outcomes, including sedentary behavior, unhealthy diets, insufficient physical activity, and inadequate sleep (6–9). Importantly, such behaviors often co-occur. For example, adolescents with poor dietary habits are more likely to also engage in excessive screen time and insufficient exercise (10–14). These overlapping behaviors highlight the need to examine health behaviors as interrelated patterns rather than isolated risk factors.

Most existing studies have used variable-centered methods, such as regression or factor analysis, which estimate the independent effects of single behaviors. While informative, these approaches overlook the heterogeneity of behavior clusters across individuals. More recently, person-centered methods such as latent class analysis (LCA) offer the ability to identify subgroups of adolescents who share distinct behavior profiles (15). This approach can better capture the complexity of co-occurring health behaviors and their differential associations with body weight. However, research applying LCA to adolescent health behaviors remains limited, and few studies have specifically investigated how these behavior patterns relate to weight status. This gap constrains the development of targeted interventions that address the needs of distinct behavioral subgroups.

Adolescence is a critical developmental stage for establishing lifelong health habits and represents a key window of opportunity for preventing obesity and related chronic diseases later in life (16–20). Understanding the heterogeneity of adolescent health behaviors and their associations with weight status is therefore essential.

To address this gap, the present study used latent class analysis to identify health behavior patterns among adolescents and examined their associations with body weight status. By clarifying how different behavioral clusters relate to risks of underweight, overweight, and obesity, this study aims to provide evidence to inform precision health management strategies tailored to the behavioral characteristics of adolescent subgroups.

## Methods

### Study design and population

We conducted a cross-sectional study among junior high school students in grades 7 and 8 from Lin'an District, Hangzhou, China, and reported our findings in accordance with the Strengthening the Reporting of Observational Studies in Epidemiology (STROBE) guidelines. We focused on this group because early adolescence is a critical developmental stage when health behaviors begin to solidify and weight status changes rapidly. In addition, students in these grades share comparable school environments and curricula, which enhances consistency across participants. Finally, these grades were most accessible during the data collection period, providing a feasible and representative sample for this study. Using a multistage stratified cluster random sampling approach, we selected four representative schools from the district's 12 public schools, stratified by urban–rural location and school size. Within each school, two classes per grade (grades 7–8) were randomly chosen, and all students in the selected classes were invited to participate.

Eligibility criteria included: (1) being a registered student in grades 7–8, (2) provision of written informed consent from guardians, and (3) clearance from school health staff confirming no severe physical illnesses (e.g., cardiovascular or metabolic disorders). Students with prolonged absences (≥1 month) or diagnosed intellectual/developmental disabilities were excluded.

A total of 1,212 adolescents completed the survey, exceeding the minimum required sample size, which was calculated based on an expected overweight/obesity prevalence of 15% (α = 0.05, power = 0.90, margin of error ±3%), accounting for design effect and non-response.

### Questionnaire development and evaluation

Data were collected using a self-developed questionnaire. The design was informed by relevant domestic and international research and adapted from multiple established health behavior measurement tools, tailored to the study objectives and the characteristics of the target population.

The questionnaire covered several core dimensions, including mental health, sleep quality, dietary behavior (e.g., fruit and vegetable intake, consumption of high-sugar and high-fat foods), physical activity, responses to stress (academic and family relationship stress), and other health-related behaviors (e.g., screen time). Most items were structured as single-choice or frequency-based questions assessing specific behaviors. In addition, standardized scales were incorporated to assess sleep quality and mental health (the Patient Health Questionnaire, PHQ). These scale-based items primarily used a 5-point Likert response format (“never” to “always”).

Reliability and validity assessments focused on the questionnaire's scale-based sections, which showed satisfactory psychometrics: Cronbach's α exceeded 0.65 across all dimensions and reached 0.82 overall, indicating excellent internal consistency (21). For construct validity, R-based exploratory factor analysis (EFA) yielded a KMO value of 0.87, significant Bartlett's test (*x*^2^ = 3,601.278, df = 28, *P* < 0.001), all item loadings >0.40, and a two-factor solution explaining 52.4% variance—these results together support good construct validity (22) (Supplementary Tables S1–S3).

### Data collection and processing

The survey was conducted using a standardized anonymous self-administered questionnaire with strict quality control. All field investigators completed a 12-h structured training program covering standardized administration, ethical principles in adolescent research, and strategies for addressing participant queries. During data collection, trained staff supervised implementation while minimizing external disruptions. Quality assurance included: (1) real-time completeness checks by field supervisors, (2) environmental controls to reduce distractions, and (3) random telephone verification of 10% of respondents. These procedures resulted in a 99.1% valid response rate, exceeding conventional standards for school-based surveys (23).

Questionnaire responses were entered into EpiData 3.1 using double data entry and verification to ensure accuracy, then exported to Excel and analyzed in R. All hypothesis tests adopted a significance threshold of α = 0.05.

During preprocessing, numerical codes from raw data were converted into the corresponding questionnaire options. Missing values were handled systematically: (1) for items with <5% missing, multiple imputation was applied (24), (2) for key variables (e.g., BMI, primary behavioral indicators) with 5–20% missing, the expectation–maximization algorithm was used (25), and (3) cases with >20% missing data or missing all key variables were excluded (26). The final cleaned dataset was summarized by gender, grade level, age, and parental educational attainment using standardized statistical tables.

### Statistical analyses

BMI was categorized per the WHO's age- and gender-specific BMI percentile standards for 5–19-year-olds (underweight: <5th percentile; normal weight: 5th to <85th percentile; overweight: 85th to <95th percentile; obesity: ≥95th percentile), which avoids inaccuracies of adult BMI cutoffs (27).

We first conducted descriptive analyses to characterize the study population. Continuous variables were summarized as means with standard deviations, while categorical variables were expressed as frequencies and percentages (28).

To identify underlying health behavior patterns, we employed latent class analysis (LCA) using the poLCA package in R (29, 30). LCA is a probabilistic, person-centered method that classifies individuals into unobserved subgroups based on their response patterns, thereby capturing the heterogeneity of co-occurring behaviors. Variable selection for the LCA model was guided by both theoretical framework and data-driven evidence, ensuring the included indicators were conceptually meaningful and empirically associated with adolescent weight status. Theoretically, indicators were grounded in “co-occurring health behaviors and obesity etiology” (31), as adolescent weight is shaped by synergistic interrelated factors rather than single behaviors (32, 33). Dietary indicators were selected given robust evidence that these factors are closely linked to adolescent weight status—insufficient fresh fruit intake and excessive sugary drink consumption consistently correlate with increased overweight/obesity risk (34, 35). Outdoor activity duration was included based on accumulating evidence that insufficient physical activity is a key modifiable risk factor for adolescent overweight and obesity (36). Anxiety control and weight management awareness/needs drew on social cognitive theory (highlighting psychological self-efficacy in behavior practice) (37) and were validated in prior adolescent health LCA studies (38). Data-driven refinement used a multi-stage strategy: random forest first selected top 20% BMI-associated behavioral variables; logistic regression (cross-validation) and Lasso regression then eliminated multicollinearity/redundancy; finally, theoretical relevance and correlation analysis confirmed the 6 core indicators for LCA.

Model selection was guided by multiple criteria. Fit was assessed using the Akaike Information Criterion (AIC) and Bayesian Information Criterion (BIC), with lower values indicating better fit. Likelihood-based statistics (*G*^2^ and log-likelihood) were also examined, and classification accuracy was evaluated using entropy values, with scores closer to 1 reflecting more precise class separation. To evaluate model robustness, sensitivity analyses were performed by reconstructing models after systematically removing core variables. Stability was confirmed when changes in AIC and BIC did not exceed 5%, entropy fluctuations remained below 0.05, and the proportion and behavioral characteristics of the latent classes showed minimal variation. Additionally, we required that associations between latent classes and BMI classification, as estimated by logistic regression, remained consistent in direction and statistical significance (*P* < 0.05).

To enhance interpretability, covariates not directly included in the class-defining indicators were incorporated into the models (39). This approach allowed us to better characterize subgroup profiles and improve model fit. Finally, after identifying the optimal latent class solution, multivariate logistic regression models were constructed to examine (1) the associations between class membership and BMI classification, and (2) the predictive effects of covariates on class assignment. All models were assessed for multicollinearity using the variance inflation factor (VIF) and for overall fit using the Hosmer–Lemeshow test.

## Results

### Sample characteristics

A total of 1,211 adolescents (605 boys, 49.9%; 606 girls, 50.0%) were included in the analysis, with a mean age of 13.15 years (SD = 0.69). Most participants were in seventh grade (62.7%), and parental education was predominantly at the junior high school level. The largest proportion of households reported annual per capita income between 10,000 and 29,999 yuan (21.7%). Detailed demographic characteristics are presented in Table 1.

**Table 1:** Demographic characteristics of youth in Lin'an District, Hangzhou City.

**Variable**	**Option**	**Frequency**	**Proportion (%)**
Grade	7th grade	760	62.71
	8th grade	451	37.21
Gender	Male	605	49.92
	Female	606	50
Actual age	≤ 12 years old	196	16.71
	13 years old	637	52.56
	14 years old	337	27.81
	15 years old	20	1.65
Father's educational attainment	Elementary school or below	78	6.44
	Junior high school	490	40.43
	High school/vocational high school/technical secondary school	299	24.67
	College/University	166	13.70
	Master's/Doctorate	19	1.57
	Unknown	159	13.12
Mother's educational attainment	Elementary school or below	139	11.47
	Junior high school	450	37.13
	High school/vocational high school/technical secondary school	258	21.29
	College/University	165	13.61
	Master's/Doctorate	28	2.31
	Unknown	171	14.11
Annual per capita household income	Below 10,000 yuan	80	6.60
	10,000–29,999 yuan	263	21.70
	30,000–49,999 yuan	205	16.91
	50,000–79,999 yuan	214	17.66
	80,000–149,999 yuan	182	15.02
	150,000 yuan or above	98	8.09

### Latent class analysis of health behaviors

Six core behavioral indicators were retained for latent class modeling (dietary intake, outdoor activity, anxiety control, weight management cognition, and needs). Comparison of models with 2–5 classes indicated that the three-class solution provided the best balance of model fit and interpretability (AIC = 14,770.95; BIC = 15,148.36; entropy = 0.511; Table 2).

**Table 2:** LCA model running results.

**Class**	**AIC**	**BIC**	***G* ^2^**	**Log-likelihood**	**Entropy**	**Residual df**
2	14,808.75	15,058.65	1,603.354	−7,355.375	0.384	14,350
3	14,770.95	15,148.36	1,515.559	−7,311.477	0.511	14,325
4	14,755.74	15,260.64	1,450.341	−7,278.869	0.478	14,300
5	14,755.56	15,387.96	1,400.160	−7,253.778	0.333	14,275

Data Source: The author organized and compiled the data from Rstudio and created the table.

**Figure 1:**
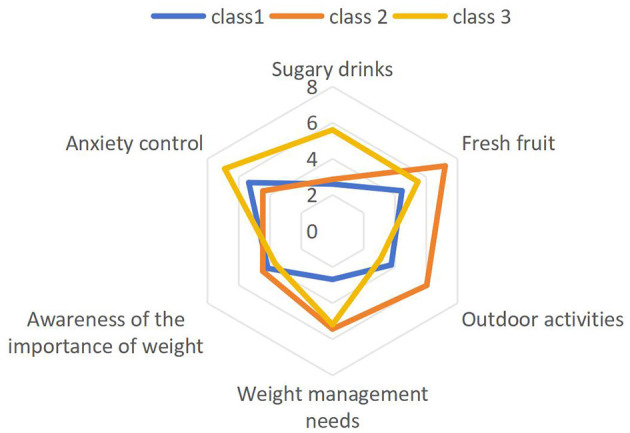
Comparison of potential category analysis characteristics.

The three latent classes were characterized as follows (Table 3; Figure 1): Class 1: passive health maintenance (50.9%)-characterized by limited fruit consumption, insufficient outdoor activity, and weak weight management. Class 2: self-disciplined health type (32.7%)-marked by balanced diet, regular physical activity, and high self-regulation. Class 3: high-risk lifestyle (16.5%)-defined by frequent intake of sugary drinks, sedentary behavior, and elevated psychological distress.

**Table 3:** Potential category analysis results.

**Variable**	**Classification**	**Class 1**	**Class 2**	**Class 3**
Fresh fruit	Rarely	0.4230	–	0.4268
	Once a day	0.5002	0.7531	0.2379
	Twice or more a day	0.0768	0.2448	0.3145
Sugary drinks	Rarely	0.9419	0.9256	0.7208
	Once a day	0.0550	0.0445	0.1826
	Twice or more a day	0.0031	0.0299	0.0916
Outdoor activities	Less than 1 h	0.2801	0.0211	0.3256
	1–2 h (excluding 2)	0.5856	0.7052	0.1543
	2–3 h (excluding 3)	0.0576	0.1755	0.2006
	3 h or more	0.0096	0.0797	0.1379
Anxiety control	None at all	0.6137	0.7148	0.4142
	A few days ( ≤ 7)	0.3041	0.1388	0.2432
	More than half the days (>7)	0.0539	0.0332	0.0982
	Almost every day	0.0277	0.0317	0.0268
Awareness of the importance of weight	Not at all important	–	–	0.0852
	Not very important	0.0199	0.0235	0.0522
	Average	0.1760	0.0989	0.2222
	Fairly important	0.4298	0.2704	0.2004
	Very important	0.3715	0.6072	0.4341
Weight management needs	Must have professional guidance	0.0141	0.0193	0.0133
	Regular supervision required	0.1795	0.0760	0.1716
	Occasional advice needed	0.5053	0.4687	0.3152
	Completely self-managed	0.2680	0.4060	0.4412

Data Source: The author organized and compiled the data from Rstudio and created the table.

### Influence of covariates on class membership

Sequential inclusion of covariates improved model fit (AIC decreased from 14,770.95 to 14,565.86). Multivariate logistic regression analyses indicated that girls were less likely than boys to belong to the high-risk class (OR = 0.53, 95% CI: 0.35–0.80). Good sleep quality was also protective (OR = 0.30, 95% CI: 0.17–0.53), whereas emotional eating increased the likelihood of belonging to the passive health maintenance group (OR = 1.74, 95% CI: 1.31–2.32; Supplementary Tables S4–S5).

### Association between health behavior patterns and BMI

After adjusting for gender, grade, and family income, latent class membership was significantly associated with BMI status (Table 4). Compared with the self-discipline group, the passive health maintenance group showed significantly increased risks of overweight (OR = 1.62, 95% CI: 1.02–2.57) and obesity (OR = 1.68, 95% CI: 1.13–2.50). The high-risk lifestyle group showed a trend toward increased obesity risk, but this did not reach statistical significance (OR = 1.57, 95% CI: 0.96–2.55, *P* = 0.072). Additional effects were observed for demographic covariates: the risk of overweight (OR = 0.56, 95% CI: 0.36–0.87) and obesity (OR = 0.41, 95% CI: 0.28–0.59) was significantly lower in females than in males. The risk of obesity in Grade 8 was significantly lower than in Grade 7 (OR = 0.59, 95% CI: 0.40–0.87).

**Table 4:** Results of multivariate logistic regression analysis of potential categories and BMI classification (including confounding variables).

**Category**	**Option**	**BMI**	**β**	**Standard error**	**OR**	**95% CI**	***P***
Potential category	Class 1 vs. Class 2	Underweight	−0.321	0.286	0.73	0.39, 1.34	0.307
		Overweight	0.483	0.234	1.62	1.02, 2.57	0.039
		Obese	0.519	0.203	1.68	1.13, 2.50	0.010
	Class 3 vs. Class 2	Underweight	0.417	0.388	1.52	0.74, 3.12	0.248
		Overweight	−0.249	0.443	0.78	0.36, 1.69	0.527
		Obese	0.451	0.300	1.57	0.96, 2.57	0.072
Gender	Female vs. Male	Underweight	0.093	0.267	1.10	0.66, 1.83	0.711
		Overweight	−0.579	0.218	0.56	0.36, 0.87	0.009
		Obese	−0.897	0.187	0.41	0.28, 0.59	<0.001
Grade	8th grade vs. 7th grade	Underweight	−0.019	0.270	0.98	0.58, 1.65	0.946
		Overweight	0.167	0.216	1.18	0.78, 1.79	0.433
		Obese	−0.525	0.192	0.59	0.40, 0.87	0.006
Annual per capita household income	Over 150,000 yuan	Underweight	0.217	0.591	1.24	0.42, 3.68	0.703
		Overweight	−0.736	0.420	0.49	0.22, 1.08	0.076
		Obese	−0.749	0.384	0.47	0.23, 0.97	0.038
	30,000–49,999 yuan	Underweight	0.964	0.446	2.62	1.13, 6.10	0.026
		Overweight	−0.971	0.366	0.38	0.19, 0.78	0.008
		Obese	0.190	0.249	1.21	0.75, 1.96	0.437
	50,000–79,999 yuan	Underweight	1.060	0.433	2.89	1.28, 6.53	0.011
		Overweight	−0.671	0.324	0.51	0.27, 0.96	0.036
		Obese	−0.426	0.277	0.65	0.38, 1.12	0.120
	80,000–149,999 yuan	Underweight	0.154	0.533	1.17	0.41, 3.33	0.771
		Overweight	−0.115	0.292	0.89	0.52, 1.53	0.673
		Obese	−0.313	0.285	0.73	0.43, 1.26	0.250

Data Source: The author organized and compiled the data from Rstudio and created the table.

### Sensitivity analysis

To assess robustness, a reduced five-variable model (excluding sugary drink frequency) was tested. Model fit improved substantially (ΔAIC = −927.91, ΔBIC = −973.82), while entropy remained stable (0.503 vs. 0.511). Class proportions and response profiles were highly consistent with the original model, and key associations with BMI remained statistically significant. For instance, the obesity risk for the passive health maintenance class persisted (OR = 1.59, 95% CI: 1.04–2.44; Supplementary Tables S6–S8). These results confirm the stability of the latent class structure and the robustness of its predictive associations.

## Discussion

This study identified three distinct health behavior patterns among Chinese adolescents in grades 7–8—passive health maintenance, self-disciplined health, and high-risk lifestyle—and demonstrated their differential associations with body weight status. Notably, the largest group, comprising over half of the sample, fell into the “passive” category, characterized by insufficient fruit consumption, limited physical activity, and weak weight management. This group, while not overtly high-risk, nevertheless faced a significantly elevated likelihood of overweight. By contrast, the “high-risk lifestyle” group, although smaller in size, exhibited a doubled risk of obesity. These findings highlight the nuanced heterogeneity of adolescent health behaviors and suggest that weight-related risks extend beyond traditionally recognized “high-risk” profiles.

Our results reinforce the view that adolescent health behaviors cluster into identifiable patterns rather than occurring in isolation. The discovery of a large “passive health maintenance” group is particularly important, as it reveals a form of “sub-health” behavior profile—not extreme enough to draw clinical or policy attention, but sufficient to increase overweight risk. This challenges the conventional focus on only the most visible or extreme risk groups and aligns with emerging evidence that moderate but persistent deficits in diet and physical activity exert significant cumulative health effects (40–42).

The passive health maintenance group presents a key behavioral paradox: basic health awareness exists but fails to translate into consistent practice, which can be explained by four interconnected mechanisms. First, academic pressure and time scarcity lead Chinese junior high students to face heavy homework and after-school tutoring, making them prioritize academics over physical activity or balanced meals. Second, environmental and social constraints—such as families lacking structured health routines, schools often replacing PE with academic courses, and little fresh fruit offered during breaks—normalize passive health behaviors. Third, these adolescents “know healthy behaviors but don't act” due to low self-efficacy. Fourth, unlike those in the high-risk group, passive adolescents face no immediate negative feedback (e.g., obvious weight gain or physical discomfort), which leads to underestimated health risks and low motivation for behavioral change.

The study also confirms and extends previous findings on gender and psychosocial correlates. Consistent with prior literature, boys were more likely to be overweight, while good sleep quality strongly predicted membership in the self-disciplined group (43). The strong link between emotional eating and the passive group further underscores the interplay between psychological regulation and health behaviors. In addition, the “dual burden” of underweight and obesity observed in adolescents from middle-income households suggests that nutritional challenges in this group are complex and cannot be explained by simple economic gradients, reflecting unique sociocultural and dietary transitions in China (44).

The behavioral patterns identified in this study suggest underlying mechanisms worth noting. The “passive” group may reflect behavioral inertia, where basic health awareness exists but is insufficiently translated into practice due to low self-regulation or environmental constraints (e.g., academic load, family habits) (45). This aligns with the construct of self-efficacy in social cognitive theory, emphasizing the gap between knowledge and action. In contrast, the “high-risk lifestyle” group illustrates a behavioral-psychological double burden: poor dietary habits co-occurring with psychological distress, potentially reinforcing a cycle of stress-driven unhealthy behaviors and elevated obesity risk. These mechanistic interpretations provide a framework for tailoring interventions beyond simple health education.

The identification of three distinct behavioral groups has direct implications for adolescent weight management, particularly for the large passive health maintenance group. Based on the aforementioned mechanisms, a school-family collaborative intervention framework is proposed. At the school level, support measures include integrating 10-min “micro-physical activities” (such as brisk walking and stretching) between classes to ensure daily exercise without disrupting academic schedules, launching a “fresh fruit program” that provides free or subsidized fruits (like apples and bananas) during breaks to lower barriers to healthy eating, and revising PE curricula to replace repetitive exercises with fun, skill-based activities that boost student engagement; at the family level, modification strategies involve holding parent workshops focused on “low-effort health routines” (e.g., adding one vegetable to each meal, taking 20-min evening walks with children), providing families with health trackers to record fruit intake and outdoor activity time for enhanced shared accountability, and encouraging parental role modeling—such as reducing sugary drink consumption and joining children in physical activities—to normalize healthy behaviors. For the passive group specifically, prioritizing the establishment of consistent health routines is key to translating their existing basic health awareness into sustained healthy practices.

For the smaller but more vulnerable high-risk group, multi-component interventions that integrate nutritional counseling, physical activity promotion, and psychological support (e.g., cognitive-behavioral therapy for stress and emotional regulation) are required. At the policy level, our findings support the integration of health behavior screening tools into school health systems, enabling early identification of risk groups through brief, reliable indicators. Differentiated strategies—progressive goals for passive groups and integrated services for high-risk groups—may improve the effectiveness of adolescent obesity prevention.

This study makes several methodological contributions. First, the use of a multi-stage sampling strategy and a large, balanced sample provides robust statistical power. Second, the combination of machine learning methods (random forest, Lasso) and theoretical considerations ensured rigorous variable selection for latent class analysis. Third, the reliability and validity of the measurement tools were systematically evaluated, strengthening confidence in the findings.

Nonetheless, some limitations should be acknowledged. The cross-sectional design precludes causal inference, and future longitudinal studies are needed to confirm temporal relationships between behavior patterns and weight outcomes. The sample was limited to one urban district in China, which may restrict generalizability to rural or culturally diverse populations. Finally, the reliance on self-reported measures introduces potential recall and social desirability biases, although the use of standardized administration and quality control likely mitigated these effects.

In conclusion, by uncovering a large but underrecognized group of adolescents with “sub-healthy” behaviors that elevate overweight risk, this study contributes a new perspective to the literature on adolescent obesity. It demonstrates that risk is not confined to the most extreme lifestyles, but also emerges from seemingly moderate yet insufficient health practices. The findings underscore the importance of considering behavioral heterogeneity in designing public health strategies and call for precision interventions that address both overt high-risk groups and less visible but highly prevalent passive groups. Ultimately, this work advances the understanding of the complex behavioral foundations of adolescent weight status and provides a practical framework for targeted prevention.

## Data Availability

The original contributions presented in the study are included in the article/Supplementary material, further inquiries can be directed to the corresponding authors.
