# Correction: Adolescent health behavior patterns and weight status: a cross-sectional analysis

**DOI:** 10.3389/fpubh.2026.1778588

**Published:** 2026-02-17

**Authors:** Jiayan Gui, Hui Zhang, Jingyou Miao, Xinyao Liu, Lilu Ding, Qiuli Wang

**Affiliations:** 1 Zhejiang Medical & Health Group Quzhou Hospital, Quzhou City, Zhejiang Province, China; 2 Department of Epidemiology and Health Statistics, Hangzhou Medical College, Hangzhou, China

**Keywords:** adolescent health behaviors, latent class analysis (LCA), body weight status, BMI classification, health intervention

Affiliation “Zhejiang Medical & Health Group Quzhou Hospital, Quzhou City, Zhejiang Province, China” was erroneously given as “Affiliated Zhejiang Medical & Health Group Quzhou Hospital, Hangzhou Medical College, Quzhou, China”.

Affiliation “Zhejiang Medical & Health Group Quzhou Hospital, Quzhou City, Zhejiang Province, China” was omitted for author Jiayan Gui. This affiliation has now been added for author Jiayan Gui.

In the published article the affiliations were included in the wrong order. The correct order appears below:

^1^Zhejiang Medical & Health Group Quzhou Hospital, Quzhou City, Zhejiang Province, China,

^2^Department of Epidemiology and Health Statistics, Hangzhou Medical College, Hangzhou, China

The corresponding author emails were incorrect in the published article. The correct emails for the corresponding authors are, Lilu Ding, I.ding@hmc.edu.cn, Qiuli Wang, Wqqxb1013@163.com.

The reference for 6 was erroneously written as “Bureau of Disease Control and Prevention National Health Commission of the People's Republic of China. Report on Nutrition and Chronic Diseases of Chinese Residents. Beijing: People's Medical Publishing House (2020).” It should be “Martin A, Booth JN, Laird Y, Sproule J, Reilly JJ, Saunders DH. Physical activity, diet and other behavioural interventions for improving cognition and school achievement in children and adolescents with obesity or overweight. *Cochrane Database Syst Rev*. (2018) 3:CD009728. doi: 10.1002/14651858.CD009728.pub4”

The reference for 9 was erroneously written as “CDC. Youth risk behavior surveillance, U.S. 2007. MMWR MorbMortal Wkly Rep. (2008) 57:1–131.” It should be “CDC. Youth risk behavior surveillance, U.S. 2007. *MMWR Morb Mortal Wkly Rep*. (2008) 57:1–131. Available online at: https://www.cdc.gov/mmwr/preview/mmwrhtml/ss5704a1.htm (Accessed December 31, 2025).”

The reference for 23 was erroneously written as “Jones RM. et al. Methodological considerations for school-based survey research: achieving high response rates in adolescent populations. J Adolesc Health. Q18 (2021) 68:456–62.” It should be “Zhao ZR, Zeng FJ, Ren WJ, Song YL, Tian J, Li P. Mental health status of primary school students during the coronavirus disease 2019 epidemic and its influencing factors. *Zhongguo Dang Dai Er Ke Za Zhi*. (2021) 23:626–32. Chinese. doi: 10.7499/j.issn.1008-8830.2012035”

The reference for 28 was erroneously written as “Daniel WW. Biostatistics: A Foundation for Analysis in the Health Sciences, 10thEdn. Wiley (2013).” It should be “Daniel WW. *Biostatistics: A Foundation for Analysis in the Health Sciences, 10th Edn*. Wiley (2013). Available online at: https://faculty.ksu.edu.sa/sites/default/files/145_stat_-_textbook.pdf (Accessed December 31, 2025).”

The reference for 40 was erroneously written as “World Health Organization (WHO). Diet, nutrition and the pre-vention of chronic diseases. World Health Organ Tech Rep Ser. (2003) 916:i−149.” It should be “World Health Organization (WHO). *Diet, Nutrition and the Prevention of Chronic Diseases*. World Health Organization Technical Report Series 916 (2003). p. i-149. Available online at: https://iris.who.int/server/api/core/bitstreams/c4f12939-5f55-4b75-b6f9-283024956b3f/content (Accessed December 31, 2025).”

The reference for 42 was erroneously written as “Kwon S, Janz KF, Letuchy EM, Burns TL, Levy SM. Association between body mass index and health-related quality of life of children and adolescents: evidence from a population-based study. J Pediatr. (2017) 190:60–7.” It should be “Ul-Haq Z, Mackay DF, Fenwick E, Pell JP. Meta-analysis of the association between body mass index and health-related quality of life among children and adolescents, assessed using the pediatric quality of life inventory index. *J Pediatr*. (2013) 162:280–6.e1. doi: 10.1016/j.jpeds.2012.07.049”

The original version of this article has been updated.

