# Supplementary material for: Adolescent health behavior patterns and weight status: a cross-sectional analysis
Source: Front Public Health. 2025 Dec 18;13:1697321. doi: 10.3389/fpubh.2025.1697321 (PMC12756493; doi:10.3389/fpubh.2025.1697321)
Supplement: Supplementary file 1 [file Table_1.docx]

**Appendix**

**Table S 1 Reliability Analysis Results**

| **Dimension** | **Items** | **Cronbach's α Coefficient** |
| --- | --- | --- |
| Sleep Quality | 4 | 0.69 |
| Mental Health | 4 | 0.89 |

Data Source: The author organized and compiled the data from Rstudio and created the table.

**Table S 2 KMO and Bartlett's sphericity test results**

| **KMO measure** |  | **0.87** |
| --- | --- | --- |
| Bartlett's sphericity test | Approximate chi-square | 3601.278 |
|  | df | 28 |
|  | P. | 0.000 |

Data Source: The author organized and compiled the data from Rstudio and created the table.

**Table S 3 Exploratory Factor Analysis**

| **Measurement Items** | **Factor 1 (PA1)** | **Factor 2 (PA2)** |
| --- | --- | --- |
| Lack of motivation or interest in doing things | 0.731 | - |
| Feeling down, depressed, or hopeless | 0.798 | - |
| Feeling tense, anxious, or irritable | 0.791 | - |
| Unable to stop or control worrying | 0.758 | - |
| How do you rate the quality of your sleep at night? | - | 0.695 |
| Do you feel sleep-deprived or fatigued? | - | 0.610 |
| Do you frequently wake up during the night? | - | 0.505 |
| Do you feel that school/family activities affect your sleep? | - | 0.451 |
| SS loadings | 2.559 | 1.632 |
| Proportion Var | 32.0% | 20.4% |
| Cumulative Var | 32.0% | 52.4% |

Data Source: The author organized and compiled the data from Rstudio and created the table.

**Table S 4 Results of LCA model with covariates**

| **Category 3** | **AIC** | **BIC** | **Log-likelihood** |
| --- | --- | --- | --- |
|  | 14770.95 | 15148.36 | -7311.477 |
| **Covariates** | **AIC** | **BIC** | **Log-likelihood** |
| Gender | 14729.72 | 15127.53 | -7286.862 |
| Grade | 14766.04 | 15163.84 | -7305.019 |
| Annual Per Capita Household Income | 14749.72 | 15188.32 | -7288.861 |
| Gender + Grade + Annual Per Capita Household Income | 14735.79 | 15215.19 | -7273.896 |
| Do you change your diet due to emotional fluctuations? | 14709.14 | 15117.14 | -7274.568 |
| How do you rate the quality of your sleep at night? | 14684.5 | 15092.5 | -7262.25 |
| How do you feel about your current academic stress? | 14721.63 | 15139.83 | -7278.815 |
| Emotional eating + sleep + stress | 14565.86 | 15045.26 | -7188.929 |

Data Source: The author organized and compiled the data from Rstudio and created the table.

**Table S 5 Results of Multivariate Logistic Regression Analysis for Potential Categories and Covariates**

| **Variable Category** | **Variable Level** | **Class1 vs Class2** | | **Class3 vs Class2** | |
| --- | --- | --- | --- | --- | --- |
|  |  | OR (95%CI) | P | OR (95%CI) | P |
| Intercept | - | 1.09 (0.51–2.30) | 0.828 | 0.75 (0.24–2.34) | 0.620 |
| Gender | Female | 1.16 (0.89–1.49) | 0.266 | 0.53 (0.35–0.80) | 0.003 |
| Grade | 8th grade | 1.17 (0.90–1.53) | 0.247 | 0.71 (0.46–1.09) | 0.118 |
| Family income (<10,000 reference) | 30,000–49,999 | 1.04 (0.69–1.57) | 0.856 | 1.40 (0.74–2.66) | 0.298 |
|  | 50,000-79,999 | 0.73 (0.49–1.09) | 0.120 | 0.62 (0.31–1.23) | 0.172 |
|  | 80,000-149,999 | 0.75 (0.49–1.13) | 0.166 | 0.74 (0.37–1.48) | 0.395 |
|  | 150,000+ | 0.62 (0.37–1.05) | 0.074 | 1.40 (0.67–2.91) | 0.369 |
| Emotional eating (never) | Often/always | 1.08 (0.75–1.57) | 0.671 | 1.66 (0.97–2.84) | 0.065 |
|  | Occasionally | 1.74 (1.31–2.32) | <0.001 | 1.01 (0.61–1.66) | 0.973 |
| Sleep quality (poor reference) | Good/very good | 0.60 (0.40–0.91) | 0.017 | 0.30 (0.17–0.53) | <0.001 |
|  | Average | 0.85 (0.56–1.30) | 0.457 | 0.53 (0.30–0.95) | 0.031 |
| Academic stress (no stress reference) | Moderate/severe stress | 1.60 (0.87–2.94) | 0.133 | 1.62 (0.61–4.28) | 0.329 |
|  | Average | 1.31 (0.73–2.36) | 0.361 | 0.89 (0.34–2.31) | 0.812 |

Data Source: The author organized and compiled the data from Rstudio and created the table.

**Table S 6 Comparison of LCA results between the two models**

| **Indicator** | **Original Model (6 variables)** | **Reduced Model (5 variables)** | **Change Magnitude** |
| --- | --- | --- | --- |
| AIC | 14,770.95 | 13,843.04 | ↓927.91 |
| BIC | 15,148.36 | 14,174.54 | ↓973.82 |
| G² | 1,515.56 | 989.87 | ↓525.69 |
| Entropy | 0.511 | 0.503 | ↓0.008 |

Data Source: The author organized and compiled the data from Rstudio and created the table.

**Table S 7 Results of latent class analysis after removing variables**

| **Variable** | **Classification** | **Class1** | **Class2** | **Class3** |
| --- | --- | --- | --- | --- |
| Fresh fruit | Rarely | 0.4109 | 0.0924 | 0.3776 |
|  | Once a day | 0.4849 | 0.7033 | 0.2925 |
|  | Twice or more a day | 0.1042 | 0.2020 | 0.3075 |
| Outdoor activities | Less than 1 hour | 0.3109 | - | 0.3778 |
|  | 1-2 hours (excluding 2) | 0.5498 | 0.7712 | - |
|  | 2-3 hours (excluding 3) | 0.0527 | 0.1545 | 0.2481 |
|  | 3 hours or more | 0.0068 | 0.0634 | 0.1815 |
| Anxiety control | None at all | 0.5564 | 0.7373 | 0.4806 |
|  | A few days (≤7) | 0.3349 | 0.1271 | 0.2239 |
|  | More than half the days (＞7) | 0.0712 | 0.0274 | 0.0703 |
|  | Almost every day | 0.0317 | 0.0334 | 0.0081 |
| Weight management needs | Must have professional guidance | 0.0220 | 0.0134 | 0.0010 |
|  | Regular supervision required | 0.1910 | 0.0908 | 0.1315 |
|  | Occasional advice needed | 0.5019 | 0.4636 | 0.3276 |
|  | Completely self-managed | 0.2562 | 0.3942 | 0.4842 |
| Awareness of the importance of weight | Not at all important | 0.0025 | - | 0.0883 |
|  | Not very important | 0.0301 | 0.0206 | 0.0294 |
|  | Average | 0.1860 | 0.0991 | 0.2222 |
|  | Fairly important | 0.4479 | 0.2706 | 0.1669 |
|  | Very important | 0.3334 | 0.6053 | 0.4875 |

Data Source: The author organized and compiled the data from Rstudio and created the table.

**Table S 8 Results of latent class analysis after removing variables**

| **Category** | **Option** | **BMI** | **β** | **Standard Error** | **OR** | **95% CI** | **P** |
| --- | --- | --- | --- | --- | --- | --- | --- |
| Potential Category | class1 vs.class2 | Underweight | -0.751 | 0.309 | 0.47 | (0.24, 0.92) | 0.027 |
|  |  | Overweight | 0.363 | 0.225 | 1.44 | (0.94, 2.20) | 0.095 |
|  |  | Obese | 0.468 | 0.195 | 1.59 | (1.04, 2.44) | 0.032 |
|  | class3 vs.class2 | Underweight | 0.706 | 0.376 | 2.03 | (1.00, 4.12) | 0.049 |
|  |  | Overweight | -0.107 | 0.441 | 0.90 | (0.40, 2.02) | 0.799 |
|  |  | Obese | 0.465 | 0.311 | 1.59 | (0.96, 2.63) | 0.072 |
| Gender | Female vs. Male | Underweight | 0.107 | 0.270 | 1.11 | (0.66, 1.86) | 0.702 |
|  |  | Overweight | -0.544 | 0.217 | 0.58 | (0.38, 0.89) | 0.012 |
|  |  | Obese | -0.872 | 0.187 | 0.41 | (0.28, 0.59) | <0.001 |
| Grade | 8th grade vs. 7th grade | Underweight | 0.023 | 0.271 | 1.02 | (0.61, 1.72) | 0.931 |
|  |  | Overweight | 0.182 | 0.216 | 1.20 | (0.79, 1.81) | 0.404 |
|  |  | Obese | -0.515 | 0.192 | 0.59 | (0.40, 0.88) | 0.009 |
| Annual per capita household income | Over 150,000 yuan | Underweight | 0.188 | 0.592 | 1.21 | (0.40, 3.64) | 0.737 |
|  |  | Overweight | -0.782 | 0.418 | 0.46 | (0.21, 1.02) | 0.057 |
|  |  | Obese | -0.766 | 0.384 | 0.46 | (0.22, 0.96) | 0.038 |
|  | 30,000-49,999 yuan | Underweight | 0.972 | 0.448 | 2.64 | (1.13, 6.17) | 0.024 |
|  |  | Overweight | -0.980 | 0.365 | 0.38 | (0.18, 0.78) | 0.008 |
|  |  | Obese | 0.197 | 0.248 | 1.22 | (0.75, 1.98) | 0.423 |
|  | 50,000-79,999 yuan | Underweight | 1.076 | 0.436 | 2.93 | (1.29, 6.64) | 0.010 |
|  |  | Overweight | -0.677 | 0.324 | 0.51 | (0.27, 0.96) | 0.037 |
|  |  | Obese | -0.428 | 0.277 | 0.65 | (0.38, 1.12) | 0.120 |
|  | 80,000-149,999 yuan | Underweight | 0.152 | 0.535 | 1.16 | (0.41, 3.30) | 0.782 |
|  |  | Overweight | -0.125 | 0.292 | 0.88 | (0.51, 1.51) | 0.659 |
|  |  | Obese | -0.316 | 0.285 | 0.73 | (0.43, 1.24) | 0.262 |

Data Source: The author organized and compiled the data from Rstudio and created the table.
